# Cold acclimation wholly reorganizes the *Drosophila melanogaster* transcriptome and metabolome

**DOI:** 10.1038/srep28999

**Published:** 2016-06-30

**Authors:** Heath A. MacMillan, Jose M. Knee, Alice B. Dennis, Hiroko Udaka, Katie E. Marshall, Thomas J. S. Merritt, Brent J. Sinclair

**Affiliations:** 1 Department of Biology, University of Western Ontario, London, ON, Canada; 2 Department of Chemistry and Biochemistry, Laurentian University, Sudbury, ON, Canada; 3 Landcare Research, Auckland, New Zealand; 4 Allan Wilson Centre for Molecular Ecology and Evolution, Auckland, New Zealand

## Abstract

Cold tolerance is a key determinant of insect distribution and abundance, and thermal acclimation can strongly influence organismal stress tolerance phenotypes, particularly in small ectotherms like *Drosophila*. However, there is limited understanding of the molecular and biochemical mechanisms that confer such impressive plasticity. Here, we use high-throughput mRNA sequencing (RNA-seq) and liquid chromatography – mass spectrometry (LC-MS) to compare the transcriptomes and metabolomes of *D. melanogaster* acclimated as adults to warm (rearing) (21.5 °C) or cold conditions (6 °C). Cold acclimation improved cold tolerance and led to extensive biological reorganization: almost one third of the transcriptome and nearly half of the metabolome were differentially regulated. There was overlap in the metabolic pathways identified via transcriptomics and metabolomics, with proline and glutathione metabolism being the most strongly-supported metabolic pathways associated with increased cold tolerance. We discuss several new targets in the study of insect cold tolerance (e.g. dopamine signaling and Na^+^-driven transport), but many previously identified candidate genes and pathways (e.g. heat shock proteins, Ca^2+^ signaling, and ROS detoxification) were also identified in the present study, and our results are thus consistent with and extend the current understanding of the mechanisms of insect chilling tolerance.

Low temperature tolerance is a key determinant of insect distribution, because the physiological effects of temperature ultimately determine physical performance and reproductive success^1^,^2^,^3^,^4^. The extensive inter- and intra-specific variation in *Drosophila* thermal tolerance makes the genus a useful model for linking the effects of temperature on biochemistry and physiology to ecological patterns and processes^5^,^6^,^7^,^8^. Because of its cosmopolitan distribution in the wild, large degree of thermal tolerance plasticity^8^,^9^,^10^, and status as a model organism^11^,^12^,^13^, *D. melanogaster* Meigen is at the forefront of these investigations. Like many insects, *Drosophila* enter a state of paralysis termed chill coma at their critical thermal minimum (CT_min_)^14^,^15^,^16^,^17^. As chill-susceptible insects, adult flies lose ion and water balance during cold exposure, which eventually leads to cell death, tissue damage, physical performance deficits, and mortality^17^,^18^,^19^. However, if substantial injury has not yet occurred, flies recover the ability to move when returned to favorable temperatures^20^. The response to cold in *D. melanogaster* is phenotypically plastic^6^,^9^, labile to artificial selection^21^,^22^, and segregates genetically^11^. *Drosophila* populations from higher latitudes are generally more cold-tolerant (defined as improvement in any cold tolerance trait)^23^,^24^,^26^,^27^, and there is a wealth of among-species variation in thermal tolerance within the *Drosophila* genus that persists when species are reared under common thermal conditions^5^,^25^. These evolved differences in cold tolerance appear, like phenotypic plasticity, to be partially driven by modifications in hemolymph ion and metabolite composition^5^,^18^,^28^, adaptive shifts in membrane composition that maintain membrane fluidity at low temperatures^29^,^30^, and an ability to maintain metabolic homeostasis in the face of chilling stress^31^.

Surprisingly, in spite of this wealth of phenotypic information, the molecular mechanisms underlying cold tolerance of *D. melanogaster* are not fully understood, and relatively few candidate genes or pathways have been linked directly to chilling tolerance. Quantitative trait loci (QTL) analyses of chill coma recovery time (CCRT) have only identified three QTLs associated with faster CCRT^12^,^32^,^33^, while microarray and RNA-seq studies have identified few genes consistently associated with cold tolerance or upregulated following cold exposure in *D. melanogaster*^34^,^35^. Although adult acclimation induces such large shifts in thermal tolerance phenotypes, one proteomic study identified a handful of proteins (largely muscle- and reproduction-related) that differed from controls (25 °C) after five days acclimation at 11 °C^36^. Making direct biochemical links between these existing candidates and cold tolerance phenotypes has also proven difficult. For example, *Frost* appears to encode a disordered protein^37^, is consistently upregulated after cold exposure^34^,^37^,^38^,^39^,^40^,^41^, is present in a QTL associated with chill coma^32^, and shows geographic variation in amino acid sequence consistent with environmental variation^42^. However, RNAi knockdown of Frost expression does not consistently impair cold tolerance^38^,^43^. Thus, although there is substantial cold tolerance plasticity in *D. melanogaster,* this plasticity is associated with strikingly few candidate molecules.

It is possible that this mismatch between phenotype and candidate genes has arisen because most studies have focused on the response to a single brief sub-lethal cold exposure (e.g.^34^,^35^,^41^) or compared the basal transcriptome among flies with detectable, but often small, phenotypic differences (e.g.^12^). For example, several gene-expression studies have attempted to elucidate the mechanisms underlying the rapid cold-hardening response, which is now thought to be mediated by non-transcriptomic cell signaling^44^. By contrast, longer-term cold acclimation over a period of days or weeks dramatically improves cold tolerance in almost all insects, including *Drosophila*^18^,^26^,^45^, and this robust response occurs over a timescale that is consistent with modifications to gene expression and (consequently) metabolic pathways. We postulated that comprehensively identifying changes in the transcriptome and metabolome associated with these large acclimation-induced changes in cold tolerance phenotype would allow us to identify a well-supported suite of candidate genes and pathways, allowing us to strengthen or question existing hypotheses, and generate new hypotheses of the mechanisms underlying variation in cold tolerance.

Here, we compare *D. melanogaster* adults acclimated for six days at 21.5 and 6 °C, which leads to a 2.5 °C shift in CT_min_ in this population (from 3.4 ± 0.2 to 0.9 ± 0.1 °C^5^). We compare the transcriptomic and metabolomic effects of acclimation temperature, and show that almost one third of the transcriptome and nearly half of the metabolome are differentially regulated with thermal acclimation. Importantly, we found considerable overlap in the metabolic pathways identified via transcriptomics and metabolomics, which suggests that our candidate genes, metabolites, and metabolic pathways for chilling tolerance are particularly robust. In particular, our data suggest strong roles for glutathione and arginine and proline metabolism in cold acclimation, as these pathways were enriched in both the transcriptome and metabolome. A majority of our candidate genes and pathways are also consistent with our current understanding of the physiology underlying insect chilling tolerance. For example, immune-responsive genes and genes coding for heat shock proteins and Ca^2+^ binding proteins were differentially expressed, and thus our results both support and extend the hypothesized mechanisms of insect chilling tolerance.

## Results and Discussion

### Thermal acclimation shifts the *D. melanogaster* transcriptome

**Figure 1:**
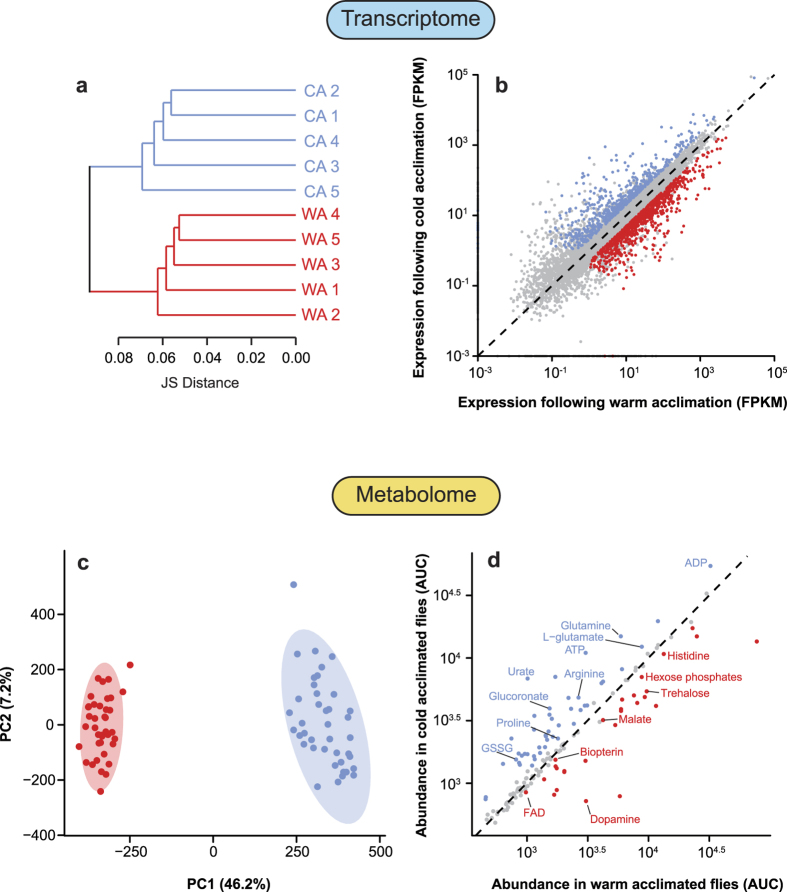
Overview of the effects of thermal acclimation on gene expression (a,b) and metabolite profiles (c,d) of *D. melanogaster*. **(a)** Dendrogram of gene expression similarity among biological replicates based on Jensen-Shannon distance of log_10_-transformed fragments per kilobase of exon per million mapped reads (FPKM). **(b)** Biplot of gene expression. **(c)** Metabolic profiles of warm and cold acclimated flies visualized with principal component analysis (points represent scores of biological replicates). **(d)** Biplot of metabolite abundance in cold- and warm acclimated flies. Differentially expressed genes (**b)**; with 2-fold expression difference between acclimation treatments and a *Q*-value < 0.01) or significantly more abundant metabolites, in warm- and cold-acclimated flies are shown in red or blue, respectively. Genes or metabolites that did not differ in expression or abundance are shown in grey. Full lists of differentially-expressed and abundant genes and metabolites are presented in Dataset 3 and Table S1, respectively.

**Figure 2:**
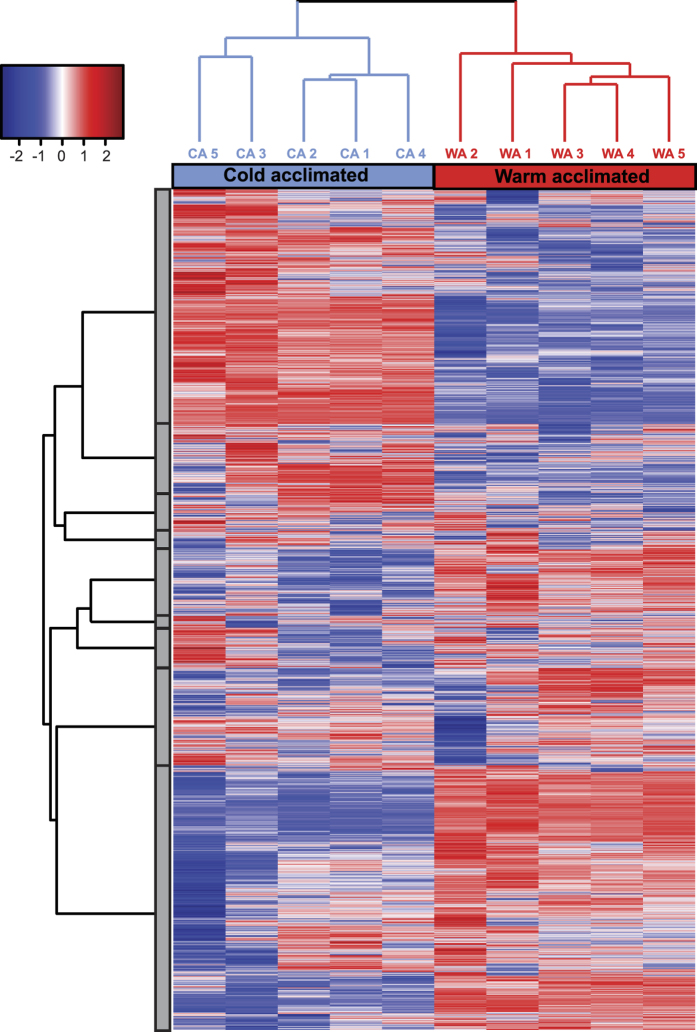
Heat map of mRNA expression profiles of male *D. melanogaster* acclimated to 6 °C (left) and 21.5 °C (right) as adults. Columns represent biological replicates (n = 5 per treatment) and rows represent individual genes. Abundance of mRNA is shown as log_2_-transformed fragments per kilobase of exon per million mapped reads (FPKM) scaled by row (gene). Transcripts with greater expression in a sample appear red and transcripts that are less abundant in a sample appear blue. Gene clustering is based on the similarity (Euclidean distance) of expression profiles across samples. Dendrograms denote the overall similarity of gene expression profiles by gene (y axis, left; limited to displaying nine large clusters shown as grey boxes), and by sample (x-axis, top). Only genes for which contigs were mapped in all ten samples are shown (n = 11,900 genes). Note that here the similarity among samples is based only on the data presented in the dendrogram, which thus differs from that presented in Fig. 1.

We examined the impact of thermal acclimation on the transcriptome of *D. melanogaster* using high-throughput mRNA sequencing (RNA-seq). An average of 11.4 and 10.5 million high-quality 50 bp reads (109 M reads in total) were mapped to the *D. melanogaster* genome from each of five biological replicates of cold- (6 °C) and warm-acclimated flies (i.e. control flies maintained at their rearing temperature of 21.5 °C), respectively (Dataset 1). A total of 11,900 genes had reads mapped to them in every one of the ten samples. Acclimation treatment heavily influenced the gene expression profile (Figs 1a and 2); a total of 4,362 genes (c. 29% of coding genes) were differentially expressed between the two acclimation temperatures based on a geometric algorithm and a false discovery rate (FDR)-corrected α cutoff of 0.05 (see methods for details). To focus our efforts on a smaller subset of (highly differentially-expressed) genes we further subset this to genes with greater than a two-fold difference in expression and *Q* < 0.01 (*P*-value following FDR), resulting in 1,567 genes that differed in expression between the two acclimation treatments (c. 10% of the genome; hereafter referred to as “differentially expressed genes”), with 649 genes upregulated and 918 downregulated following cold acclimation (Fig. 1b, Dataset 2). These numbers are orders of magnitude greater than the number of cold-tolerance candidate genes previously identified following cold exposure in *D. melanogaster*^34^,^35^, and in most other insect species^46^,^47^,^48^,^49^.

**Figure 3:**
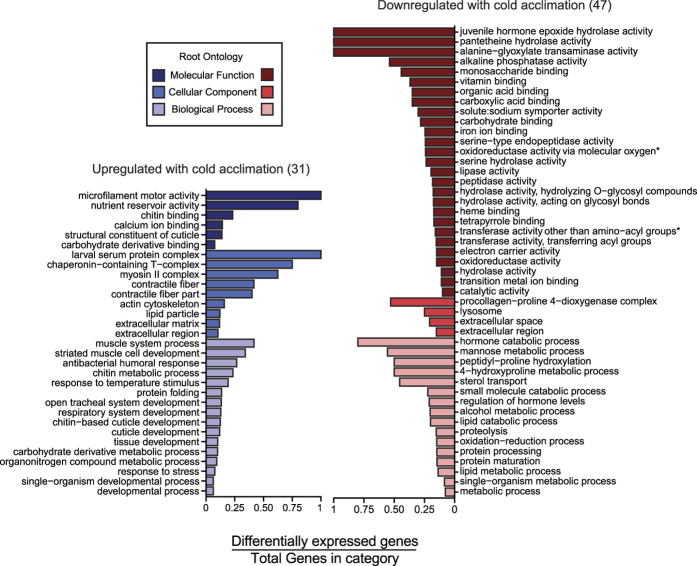
Gene Ontology (GO) terms enriched in genes upregulated with cold acclimation (blue) or downregulated with cold acclimation (i.e. higher expression in warm acclimation; red) in adult *D. melanogaster.* Bars denote the proportion of differentially expressed genes (2-fold difference in expression and a *P*-value < 0.01) relative to the total number of genes in the *D. melanogaster* genome mapped to each term. Asterisks denote terms with long titles that have been simplified for space (GO:0016705, GO:0016747). To avoid redundancy, significant GO term lists were reduced using REVIGO (see methods), but full lists of significant GO terms, including differentially expressed genes that map to each term, are presented in Dataset 3).

The majority (985; 63%) of differentially-expressed genes mapped to at least one gene ontology (GO) term, and 108 GO terms were significantly enriched in the dataset following FDR correction (Dataset 3). To simplify interpretation of this list, we used REVIGO to cluster GO terms across all differentially-expressed genes, which resulted in 49 semantically-distinct GO terms that were significantly enriched in response to acclimation (Dataset 3). We then separated genes by their direction of differential expression between the two acclimation temperatures and ran the same GO term analysis with each subset, which resulted in 31 and 47 semantically-distinct GO terms with greater expression in cold-acclimated flies and flies maintained at 21.5 °C, respectively (Fig. 3). Complete lists of significant GO terms with differentially expressed genes associated with each term can also be found in the supplementary material (Dataset 3). Several terms for cellular components and processes identified here were also enriched in the previously-sequenced transcriptomes of either *D. montana* or *D. virilis* (both in different subgenera to *D. melanogaster*) following cold acclimation (for example, cytoskeletal components, ion transporters, calcium regulation and glutathione-s-transferase activity)^48^. Despite using similar acclimation conditions to our study, Parker *et al.*^48^ associated far fewer candidate genes and GO terms with cold acclimation in these drosophilid species. This difference may arise from analytical differences between our approaches, or because the more cold-tolerant *D. montana* and *D. virilis* respond less to acclimation at this temperature. Regardless, although the GO terms associated with cold acclimation differed between *D. montana* and *D. virilis*, we observe overlap between each of these species and *D. melanogaster*, suggesting that candidate mechanisms for cold acclimation are generally conserved across the *Drosophila* phylogeny.

Several of the GO terms that were significantly enriched in cold-acclimated flies related to the muscle contractile apparatus (Fig. 3, Dataset 3), consistent with a previous proteomic survey^36^. Low temperatures strongly suppress maximal muscle force production in both fish and insects^50^,^51^, an effect that can be partially compensated for through acclimation^51^. Acclimation to low temperatures by poikilothermic fish involves reorganization of muscle fiber type and abundance, a change that is associated with differential expression of several proteins involved in muscle contraction, including the myosin light and heavy chains^52^,^53^. Our results suggest a similar reorganization of the muscle fibers during adult cold acclimation in *Drosophila*, as the genes encoding myosin heavy chain (*Mhc*) and both myosin light chains 1 and 2 (*Mlc1, Mlc2*) were upregulated 3.5-fold (Dataset 2). In addition, cold-acclimated flies upregulated genes encoding troponin C and troponin T (including *upheld, Tpn25D, Tpn41C, TpnC47D, and TpnC73F*; Dataset 3), which facilitate Ca^2+^-mediated muscle contraction. This increased transcriptional investment in the muscle systems could mitigate the observed depolarization of muscle cell resting potentials^54^,^55^ and decreased muscle force generation at low temperatures^50^. Warm-acclimated *D. melanogaster* lose the ability to fly at relatively mild low temperatures (c. 14–18 °C)^56^, and our results suggest that flies could modulate muscle force generation in response to low temperature exposure to maintain flight performance even if development at high temperatures has already fixed wing morphology^56^. Calcium regulatory proteins largely determine the thermal sensitivity of the actin-myosin complex, and are also differentially regulated during cold acclimation in fish^57^. Thus, these modifications to Ca^2+^ binding and the actin-myosin machinery could be essential to maintenance of muscle contractility at low temperatures, and could be a critical determinant of reproductive success in natural settings. Diapausing mosquitos (*Culex pipens*) have greater abundance of polymerized actin at muscle fiber intersections in the midgut^58^, and chilling disrupts cytoskeletal organization in primary embryonic cultures of *Drosophila* cells^59^. Thus, regulation of cytoskeletal function is likely to be an important component of cold acclimation in a variety of tissues beyond muscle.

Transcripts for several genes that have been previously associated with cold stress or cold tolerance in *Drosophila* were significantly upregulated in cold-acclimated flies. For example, *Frost*, *smp-30, Starvin* and *hsr-omega* transcripts were all upregulated during cold acclimation in the present study, and have all previously been associated with cold tolerance or cold exposure in *D. melanogaster*^38^,^60^,^61^,^62^,^63^,^64^. Many genes encoding heat shock proteins were upregulated in cold-acclimated flies, including transcripts for HSP22, HSP23, HSP26, HSP67, and HSP70Bbb (Dataset 4). Many of these candidate genes are also upregulated in response to a brief cold stress (e.g. 0 °C for 2h)^34^ and/or cold acclimation^65^ in *D. melanogaster*. Expression of all of these candidate cold tolerance genes is highly enriched in the Malpighian tubules^66^, which supports the notion that the renal epithelia, and the Malpighian tubules in particular, are central to insect cold tolerance^18^,^28^,^67^,^68^, and targets the tubules as a tissue in which to better determine the physiological roles of these molecules. A tissue-focused approach may help to resolve conflicting evidence about the role of these candidates in determining cold tolerance phenotypes, as whole-animal knockdowns often have no effect on cold tolerance phenotypes (e.g.^43^,^65^). The upregulation of these genes during acclimation to a mild low temperature that does not induce chill coma supports their importance in acute thermal tolerance, but suggests that their upregulation following a cold stress reflects the initiation of thermal acclimation, rather than an adaptive response to acute injury.

Cold acclimation also involved significant upregulation of genes involved in chitin metabolism and cuticle binding (e.g. *knk*, *verm*, and *serp*), as well as those associated with immunity and lipid transport. Cuticle protein-related genes are also associated with cold tolerance and may be under positive selection in stick insects^46^. Further, knockdown of chitin synthesis can disrupt the barrier functions of the midgut peritrophic matrix of flour beetles^69^, and the barrier function of the gut epithelia appear to be of great importance to insect cold tolerance^70^. Several genes that were upregulated with cold acclimation in the present study have important roles in tracheal system development and/or may influence cuticular permeability (see Dataset 3). A relationship between cold exposure and immunity has also been suggested by numerous transcriptomic studies^35^,^71^,^72^, although the nature of this relationship remains unclear^73^, particularly since cold acclimation does not appear to improve realized immunity in crickets^74^. We observed widespread upregulation of both genes encoding antimicrobial peptides (*Dro*, *Dipt*, *AttA*, and *AttC*) and those associated with immune signaling (*Thor*, *IM3*, *IM23*, *Tep4*). Cold acclimation also involved upregulation of genes coding for all of the known components of the lipid-transporting larval serum protein complex (*Lsp1α*, *Lsp1β*, *Lsp1γ*, *Lsp2*, and *CG8100;* Fig. 3, Dataset 3), suppression of which is associated with the early stages of diapause induction in the drosophilid *Chymomyza costata*^75^,^76^.

### Impacts of thermal acclimation on the *Drosophila* metabolome

**Figure 4:**
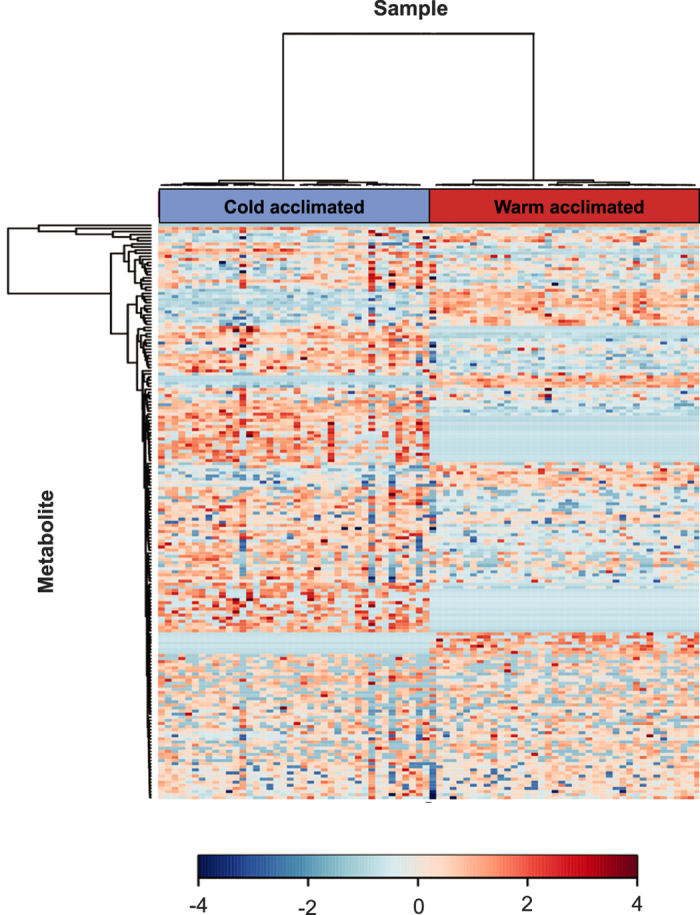
Heat map of all identifiable metabolites in male *D. melanogaster* acclimated to 6 °C (left) and 21.5 °C (right) as adults. Rows represent individual metabolites, and columns represent biological replicates (n = 40 per treatment). Samples and metabolites are grouped according to similarity, denoted by the dendrograms on the x and y-axes.

We detected and identified a broad suite of metabolites in flies from both acclimation groups (Fig. 4). The overall metabolic profiles of the acclimation groups were well-resolved in one axis of a principal component analysis (PC1), along which warm- and cold-acclimated flies were clearly resolved (Fig. 1c). A total of 187 features (potential metabolites) were detected, 90 of which (48%) significantly differed (*P* < 0.05) between flies acclimated to warm and cold conditions (Fig. 1d). We positively identified 34 (18%) features and confirmed them with standards; 26 of these known metabolites significantly differed in abundance between warm- and cold-acclimated flies (Table S1). Loadings of these confirmed metabolites along PC1 are presented in Fig. S1, and boxplots of individual metabolites are presented in Fig. S2.

**Figure 5:**
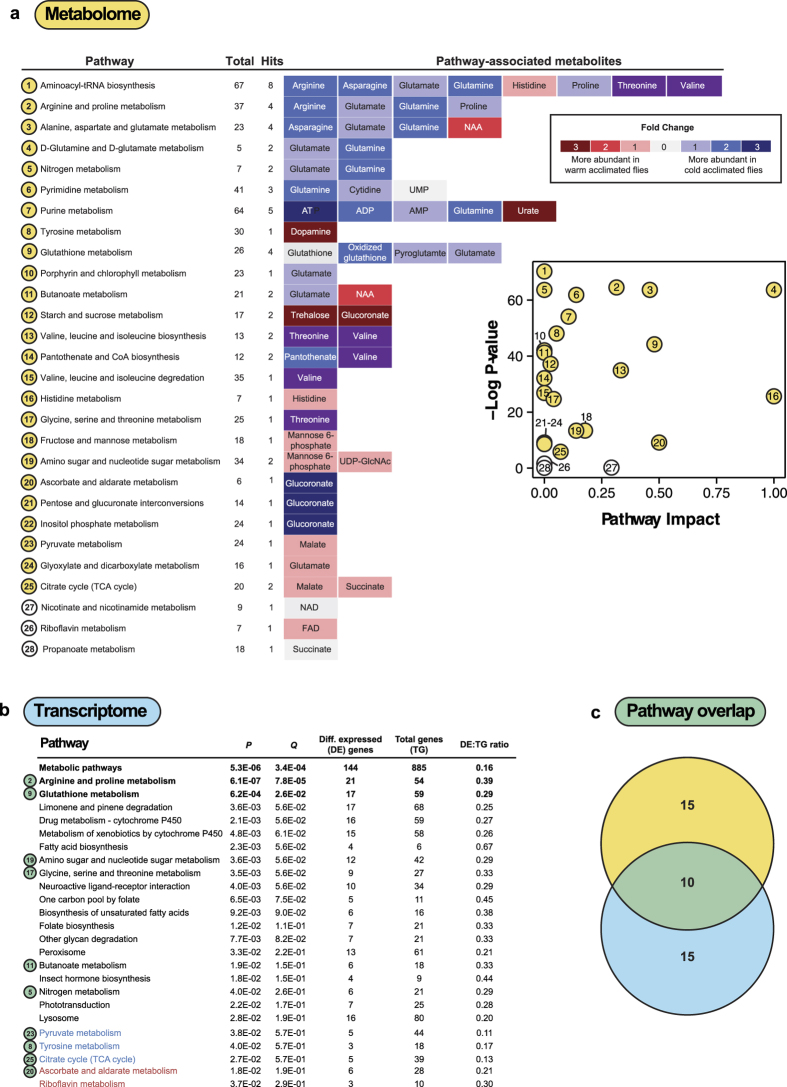
Results of KEGG pathway analysis of thermal acclimation in *D. melanogaster.* **(a)** Results of pathway analysis of identified metabolites in warm- and cold-acclimated flies. Metabolites mapped to pathways are represented along a row. Boxes surrounding metabolite names correspond to differences in the relative abundance between cold and warm acclimated flies (up-regulated in cold in blue and down-regulated in cold in red). Metabolites shown in purple were only detected in cold-acclimated flies. Total: total number of metabolites in pathway, Hits: number of metabolites in pathway that significantly differ in abundance between warm- and cold-acclimated flies. **Inset:** Statistical significance and pathway impact, with numbers corresponding to the pathways listed in panel A. **(b**) Metabolic pathways in the KEGG database that were overrepresented in genes that were significantly differentially expressed between warm- and cold-acclimated flies. Pathways listed with black text were significantly enriched when all differentially expressed genes were included in the analysis. Those shown in blue or red were significantly enriched when only genes with higher expression in cold- or warm- acclimated flies (respectively) were included in the analysis. Numbers shown in the green circles match to the same pathway shown in panel A, meaning these pathways were enriched in both analyses. Pathways shown in bold remained significant following false discovery rate correction. **(c)** Venn diagram of enriched metabolic pathways in based on differentially expressed genes and differentially abundant metabolites. Numbers correspond to the number of pathways, before FDR correction in the case of the transcriptome, that were enriched in the metabolome (yellow), transcriptome (blue) or both (green).

Of the total pool of metabolite features, 22% (41/187) were only detected in one of the two groups of flies. We identified two of these features, which were both amino acids (valine and threonine), and both were only above detectable levels in cold-acclimated flies. Several other amino acids were detected and identified in both warm- and cold-acclimated flies and were significantly more abundant in cold-acclimated flies, including arginine, asparagine, glutamine and proline (Figs 1d and 5a, Fig. S2). Interestingly, several of these amino acids have been previously reported to decrease during rapid cold-hardening in adult *D. melanogaster*^77^, but proline and asparagine increase during long-term thermal acclimation of larvae^78^, as we observed here in adults. By contrast, adult flies reared under fluctuating low temperature conditions increased valine (as observed here) but did not alter proline, glutamine, or threonine levels^79^. This variation in amino acid responses during different windows of development and reproductive maturity support the idea that rapid cold-hardening and long-term acclimation operate through different physiological mechanisms^80^, and suggest that developmental and adult acclimation have distinct metabolic outcomes.

Adenosine mono- di- and triphosphate levels were also all higher in cold-acclimated flies (Figs 1d and 5a), which suggests that an increase in the adenylate pool may be advantageous in the cold. Exposure to cold stress that causes injury and death has no effect on energy availability in the form of ATP in the fat body of firebugs^81^, and slightly increases ATP availability in the cricket muscles and beetle larvae^82^,^83^. By contrast, chronic chilling decreases whole-body ATP levels in flesh fly larvae, a detrimental state that can be rescued by interrupting the cold exposure with pulses of high temperature^84^. These different effects of chilling on ATP availability may be related to whether chilling more strongly affects metabolic pathways of ATP production or consumption. For example, cold acclimation reduces the amount of active Na^+^/K^+^-ATPase protein (a major contributor to total ATP consumption rates) in *D. melanogaster*^5^, and thus increased ATP availability may be a passive consequence of reduced ATP consumption, rather than an adaptive mechanism of thermal acclimation. Alternatively, cold acclimation may increase ATP in anticipation of acute stress, potentially to provide a larger ATP pool to fuel repair processes or reestablishment of homeostasis. Increased ATP supply could, for example, improve low temperature survival by facilitating increased rates of ATP-dependent ion-transport in epithelia during or following cold. However, the specific functions of increased ATP availability and the size of the total adenylate pool in cold tolerance remains to be thoroughly examined.

### Downregulation of genes and reductions in metabolite abundance with cold acclimation

Although stress tolerance studies tend to focus on increases in transcript and metabolite abundance, transcriptional downregulation of key processes may be of equal importance in setting thermal tolerance phenotypes. We observed a greater number of transcripts and approximately 1.5 times more semantically-distinct GO terms enriched in flies maintained at their rearing temperature than those acclimated to cold (meaning these transcripts and GO terms were inversely related to cold tolerance; Fig. 3). Transcripts that were less abundant with cold acclimation (i.e. enriched in warm-acclimated flies) were associated with juvenile hormone (JH) catalysis (driven by changes in juvenile hormone epoxide hydrolases; JHEH) as well as sodium-driven symporter activity, monosaccharide binding and peptidase (particularly trypsins) and hydrolase activity (Fig. 3, Dataset 3). Signaling through JH suppresses ovarian development during short day conditions in *D. melanogaster* females^85^ (note that in the present study day length was the same in both acclimation treatments), and JH promotes courtship behavior in males (which were sampled for the present study)^86^. Furthermore, JH stimulates production of antifreeze proteins in larvae of the freeze avoidant beetle (*Dendroides canadensis*)^87^. To our knowledge, the role of JH in chilling tolerance of *Drosophila* has not been studied, but *Jheh1* upregulation is associated with seasonal changes in the phenotype of *D. suzukii*^88^. On a shorter timescale, JH could be a candidate for coordinating physiological and behavioural adjustments in response to abiotic temperature, thus integrating multiple seasonal cues.

Several genes encoding proteins involved in multiple classes of sodium driven transport or exchange were also downregulated with cold acclimation (Fig. 3, Dataset 3), including those implicated in sodium:amino acid symport (e.g. *NAAT1, dmGlut*) and sodium:inorganic phosphate symport (e.g. *Picot*, *MFS10*, *MFS14*). If these changes in gene expression reflect a reduced reliance on sodium-driven symport mechanisms, such a change may be tied to previously-observed reductions in transcellular sodium gradients in cold-acclimated flies, a shift that is suggested to protect against ion balance disruption at low temperatures^18^. A transporter (*Nha1*) recently suggested to function as an H^+^-Cl^−^ cotransporter, rather than a Na^+^/H^+^ exchanger as originally suspected^89^, was also downregulated in cold-acclimated flies. This contrasts with the recent finding that knockdown of *Nha1* considerably slows chill coma recovery time of *D. melanogaster* adults^67^. Roles for acid-base balance and the thermal sensitivity of hydrogen ion transport in chilling tolerance have received little experimental attention, but could be a fruitful avenue of future research.

A total of ten of the 34 positively-identified metabolites significantly decreased in abundance following cold acclimation (i.e. were higher in flies maintained at 21.5 °C) including dopamine, trehalose and histidine (Table S1; Fig. 5a). The role of dopamine signaling in thermal preference in *D. melanogaster* has been examined in two studies using knock-out mutants, but these led to opposite conclusions about whether increases in dopamine signaling increase cold preference or warm preference^90^,^91^. Another potential explanation for the decrease we observed in dopamine concentration is that dopamine secreted by epidermal cells is oxidized into melanin^92^, and given that *Drosophila* invest in melanin production during development at low temperatures^93^, it may be that dopamine is being invested into melanin synthesis during cold acclimation^94^. Trehalose was also less abundant in cold-acclimated than warm-acclimated flies. This result is curious, given that trehalose is a well-described cryo- and osmoprotectant, and increases in abundance in response to cold shock or rapid cold-hardening treatments in *Drosophila* adults^77^, as well as during long-term cold acclimation of *Drosophila* larvae^78^. In the present study, cold acclimation also reduced levels of histidine in *D. melanogaster* (Fig. 5a), but selection for fast recovery from chill coma has been previously noted to increase histidine levels in the same species^31^.

### Transcriptomic and metabolomic evidence for metabolic pathway regulation

We used MetaboAnalyst^95^ and goseq^96^ to find metabolic pathways in the KEGG database that were significantly altered in the metabolome and transcriptome, respectively. A total of 28 KEGG pathways were enriched with the metabolites in our analysis, and 25 of these pathways were significantly modified during thermal acclimation (Fig. 5a). A total of 283 of the 1,567 differentially expressed genes (18%) from the transcriptomic analysis were represented in the KEGG database. Of these, 103 genes were more highly expressed in cold-acclimated flies, and 180 were more highly expressed in flies maintained at 21.5 °C. We mapped all differentially-expressed genes, as well as just those enriched in one or the other acclimation group, to KEGG pathways, and present results from each of these three independent analyses in Dataset 4. To maximize identification of overlapping pathways (those enriched in both the transcriptomic and metabolomic data), we pooled significant pathways from all three transcriptomic analyses (prior to FDR correction).

In total, 25 metabolic pathways from the KEGG database were significantly altered during thermal acclimation in each of the two ‘omics’ approaches. From these, ten enriched pathways were shared across the two approaches (Fig. 5b,c), and two of these ten pathways have the strongest support, given that they remained significant following false discovery rate correction of both datasets. These two pathways were 1) Arginine and Proline Metabolism and 2) Glutathione Metabolism. The observation that Arginine and Proline Metabolism genes and metabolites were so strongly enriched in both datasets fits with our current understanding of insect chilling tolerance, as proline has been repeatedly suggested to be preferentially used by the mitochondria as a metabolic fuel at low temperatures, and/or be involved in osmotic protection (at relatively low concentrations) and cryoprotection (at high concentrations) of insect tissues at low temperatures^97^,^98^,^99^. Indeed, a proline-supplemented diet under winter-acclimating conditions confers partial freeze tolerance to *D. melanogaster* larvae^98^. In the present study, cold-acclimated flies had higher levels of both proline (1.3-fold) and arginine (1.8-fold) than warm-acclimated flies, and also contained a greater proportion of glutamate (1.4-fold), a precursor to arginine and proline (Table S1). Further, 21 of the 54 genes that map to this pathway (39%) were differentially expressed during thermal acclimation (Fig. 5b). Together, these findings suggest that these polar and charged amino acids, and modulation of proline metabolism in particular, may be central to the acquisition of chilling tolerance. Curiously, however, selection for rapid recovery from chill coma downregulates proline metabolism, resulting in lower, rather than higher, levels in selected flies^31^. This discrepancy may arise because proline has different roles in chilling survival versus chill-coma recovery, or because selection for cold tolerance elicits trade-offs with neighboring pathways such as those related to redox balance. Increases in intracellular or extracellular proline protect mammalian cells against oxidative stress, through preservation of the glutathione redox environment, meaning proline biosynthesis may instead be indicative of an oxidative stress response^100^.

Glutamate is not only a metabolic precursor of arginine and proline, but also of glutathione, and Glutathione Metabolism was the second KEGG-derived pathway that had strong support for differential regulation in both the transcriptome and the metabolome. Interestingly, we found no difference in the abundance of reduced glutathione (GSH), but rather a higher abundance of oxidized glutathione (GSSG) in cold-acclimated flies, relative to flies maintained at their rearing temperature (Fig. 5a, Table S1). This apparent shift in redox state could imply that cold-acclimated flies are under greater oxidative stress at 6 °C, as cold stress and chilling injury have been associated with oxidative damage and rightward shift in the GSH:GSSG ratio, particularly in chill susceptible and freeze avoiding insect species^101^,^102^,^103^. In the transcriptome of *D. melanogaster*, we found 17/39 (29%) of genes mapping to the Glutathione Metabolism term were differentially expressed, including eight genes that encode glutathione-S-transferases (GSTs) and one “GST-like” protein coding gene (*Mgstl*), all of which were upregulated with cold acclimation (Dataset 4). Cold acclimation induces increases in GST abundance and activity in the fat body and plasma of larval Lepidoptera^104^,^105^, and GSH:GSSG balance plays important roles in cold stress and cold acclimation signaling in plants^106^, suggesting that this observed shift might serve a signaling, as well as protective, role.

### How does cold acclimation work?

Cold acclimation is thought to improve insect cold tolerance through an improved ability to maintain homeostasis and cellular integrity at low temperatures^107^. The primary mechanisms proposed to underlie this phenotypic plasticity are 1) protection against cold-induced ion and water balance disruption^18^, 2) defense against oxidative stress^102^, 3) maintenance of metabolic balance^31^, 4) refolding or stabilizing proteins^45^, 5) modifications to membrane fluidity to maintain cellular integrity^108^, and 6) inhibition of apoptosis^80^.

Our results support most, but not all, hypothesized mechanisms of chill tolerance. While the whole-animal metabolome cannot offer substantial insight into adjustments to ion and water balance, a large number of genes involved in ion homeostasis were differentially-expressed with cold acclimation. Our transcriptome and metabolome data suggest that glutathione metabolism may be important in avoiding or repairing oxidative damage at low temperatures, and the generally broad suite of changes we observed in metabolic enzyme transcripts and the metabolome with cold acclimation also supports a role for modifications to protein, lipid, and carbohydrate metabolism networks in cold tolerance. The general upregulation of chaperone proteins we observed following cold acclimation supports a role for preemptive protection against misfolded proteins at stressful low temperatures. Although our transcriptomic data indicate altered lipid metabolism during thermal acclimation, we saw little evidence to specifically suggest changes to membrane lipid metabolism specifically. Because our metabolomics data was biased toward polar metabolites, we were unable to identify membrane components to address membrane-related mechanisms directly. Finally, we did not see strong evidence in the transcriptome (perhaps excepting shifts in Ca^2+^ handling) to suggest changes to apoptotic signaling pathways during long-term adult thermal acclimation.

Our results beg one to ask why many of the molecular and biochemical changes noted here have not been previously associated with the low temperature biology of *Drosophila*. Previous studies in *Drosophila* have focused on acute responses to temperature (i.e. during or following minutes to hours in chill coma) rather than long-term acclimation to non- or less-stressful low temperatures. Thus, we hypothesize that these studies were exploring phenotypes driven by signaling or post-translational shifts that would not be readily apparent in the transcriptome, and whose immediate signature in the metabolome may also be weak. Future studies should thus be directed at better understanding modifications to thermal tolerance occurring over these longer time scales. As roles of specific epithelia and organs (such as the Malpighian tubules and gut epithelia), organ systems (such as the muscles and nerves), or body compartments (such as the hemocoel) in setting thermal tolerance phenotypes are now becoming apparent (e.g.^7^,^28^,^54^,^67^), we suggest ‘omics’ approaches can, and should, now be used in a more hypothesis-driven and tissue-specific manner than they have in the past (and in the present study). Such an approach could facilitate rapid discovery of molecular and biochemical plasticity that can be causally, rather than coincidentally, linked to tissue-specific and whole-organism thermal tolerance phenotypes.

## Conclusions

Temperature affects all biochemical reactions in an organism, and thus low temperature acclimation undoubtedly involves a large suite of molecular, biochemical and physiological adjustments across interacting and co-dependent organs and tissues. It is this sub-organismal “tuning” of function ultimately determines whole organism survival and fitness. Here, we generated a dataset that finally reflects this expected complexity; we have increased the number of candidate genes for cold tolerance to nearly a third of the genome of *D. melanogaster,* and note that c. 50% of the measured metabolome is altered during the same acclimation treatment. By combining these two approaches, we have 1) provided strong support for some previously reported mechanisms of cold acclimation (e.g. investment in heat shock proteins and proline synthesis); 2) drawn attention to other mechanisms that are suggested but understudied in insects (e.g. protection against oxidative stress, Ca^2+^ dynamics, and plasticity of the actin and myosin machinery); 3) contributed to an ongoing discussion of consistently observed but mechanistically unclear molecular responses to cold stress (e.g. *Frost*, immune response pathways); and 4) provided new pathways of study in the mechanisms of thermal acclimation (e.g. sodium-driven symport and endocrine signaling).

## Materials and Methods

### Animal husbandry

The *Drosophila melanogaster* population used in this study was derived from 35 isofemale lines caught in London and Niagara on the Lake, Ontario, Canada in 2007^109^ and was maintained at a constant 21.5 ± 0.5 °C and at 50 ± 5% relative humidity with a 13:11 h (L:D) light cycle. Flies were raised from egg to adult in 35 mL vials containing ~10 mL of banana-based food medium. Adult flies were transferred to 3.7 L plastic containers each generation for mass breeding and egg collection during which adult flies were provided with a petri dish of food topped with yeast paste (1.5:1 active yeast:water). After 24 h, the food plate was replaced with a fresh plate of food, which was left for 14 h before eggs were collected into fresh vials at a density of 75 eggs per vial.

Newly-eclosed adult males were collected under light CO_2_ anesthesia on the day of their emergence and divided randomly into two thermal acclimation groups with the same light and humidity conditions: 21.5 °C (warm-acclimated) and 6 ± 0.5 °C (cold-acclimated). Flies were left at their respective acclimation temperatures for five days to acclimate and to control for age and anaesthesia effects on cold tolerance^63^,^110^, before being snap-frozen in liquid nitrogen and held at −80 °C for later analysis of gene expression and metabolite abundance.

### Gene expression

Transcript abundance was quantified using high-throughput mRNA sequencing (RNA-seq). Frozen warm- and cold-acclimated male *D. melanogaster* (five biological replicates per acclimation temperature, each containing 25 flies) were homogenized by mortar and pestle over liquid nitrogen and then in 1 mL of TRIzol reagent. Homogenized samples were centrifuged at 12,000× *g* for 5 min at 4 °C and the bottom 200 μL (lipids and DNA) discarded. Chloroform (200 μL) was added to the tubes, samples were shaken for 15 s, and left at 22 °C for 3 min. Samples were centrifuged at 12,000× *g* for 15 min at 4 °C, the upper (aqueous) phase transferred to a new tube with 1 volume of 70% ethanol, and vortexed (30 s). For further sample purification, a 700 μL aliquot of the sample was centrifuged (8000× *g* for 15 s at 22 °C) through an RNeasy Mini spin column (Qiagen, Hilden, Germany) in a 2 mL collection tube. The effluent was discarded and the sample (bound to the column) was washed with 700 μL of RW1 buffer (Qiagen), and 2 × 500 μL of RPE buffer (Qiagen) using the same centrifuge settings. The column was placed into a new collection tube and RNase-free water (50 μL) was centrifuged through the column (8000× *g* for 1 min at 22 °C) twice to release the RNA from the column. Total mRNA was purified from RNA, and cDNA libraries were prepared using a TruSeq non-stranded mRNA preparation kit (Illumina Inc., San Diego, CA, USA) following the TruSeq RNA sample prep v2 protocol (Illumina). Briefly, mRNA was purified by poly-A selection using magnetic beads bound to poly-T oligo-nucleotides and chemically fragmented. Fragmented mRNA was reverse-transcribed into single stranded cDNA by reverse transcriptase and the RNA on the opposite strand was replaced with DNA to yield double stranded (ds) cDNA. Overhangs at the 3′ and 5′ ends of ds cDNA were cleaved and repaired by exonuclease and DNA polymerase, respectively. Illumina HiSeq2000 was used to sequence multiplexed (13–14 samples per lane, run alongside samples from two other experiments) single-end 50 bp cDNA libraries at the High Throughput Genomics Core Facility at the Huntsman Cancer Institute, University of Utah.

Transcriptome data manipulation and statistical analyses were completed in Galaxy^111^ following the analysis workflow of Trapnell *et al.*^112^. Briefly, Illumina adapter sequences were clipped from reads using FastQ_Clipper (part of the FastX Toolkit), and clipped reads were aligned to the genome of *D. melanogaster* (Ensembl build 5.25) using TopHat v.2.0.8^113^, with alignment limited to known splice junctions. Cufflinks^112^ was used to assemble and count transcript reads for each sample and Cuffmerge v.2.0.2 was used to merge assembled transcripts for all biological replicates into a single reference transcriptome^114^. To test for differential expression of genes that mapped to the transcriptome, we used CuffDiff 2, which uses a geometric-based algorithm to generate test statistics, *P*-values, and Benjamini-Hochberg^115^ false discovery rate (FDR) corrected *P*-values (*Q*-values) for each gene mapped^116^. More than 4,300 genes were significantly differentially expressed with thermal acclimation after FDR correction (*Q* < 0.05), so to reduce the number of targets we set cutoff values for the magnitude and statistical significant of differential expression. Genes that had both a 2-fold difference in expression between the acclimation groups, and a *Q*-value less than 0.01 were used in all downstream analyses and were deemed “differentially expressed genes”. Differentially expressed genes were mapped to the Gene Ontology (GO) term and Kyoto Encyclopedia of Genes and Genomes (KEGG) pathway databases using goseq in R^96^. To reduce the number of significant GO terms for plotting purposes, we used REVIGO, which eliminates semantically redundant terms^117^.

### Metabolite abundance

Metabolites were extracted from whole fly samples (n = 40 per acclimation temperature) following Knee *et al.*^118^, with minor modifications as described below. Samples consisted of groups of 15 male flies that were weighed to the nearest 0.01 mg and flash frozen in liquid nitrogen. The volume of extraction solvent used for each sample was normalized by the mass of the sample by pipetting 6.35 μL extraction solvent per mg of fly. The extraction solvent consisted of a 3:1:1 ratio of methanol:chloroform:water. Samples were homogenized with a mixer mill (Qiagen) at 30 Hz for 1 min. Homogenates were placed at −20 °C for 60 minutes to promote metabolite extraction and increase protein precipitation. The samples were subsequently centrifuged at 16000 RCF to remove protein and debris, and the supernatant was stored at −80 °C for later analysis.

Metabolomic analysis was carried out as previously described^118^. Briefly, LC-MS analysis was performed on a Dionex UltiMate 3000 Rapid Separation (U)HPLC (ThermoFisher) coupled to a micrOTOF QII mass spectrometer equipped with an Electrospray Ionization source (Bruker Daltonics). Samples (10 μL) were injected onto a Kinetex 100 mm × 2.1 mm, 1.7 μm C18 column (Phenomenex). Metabolites were separated using a multi-step, two solvent gradient with diamylammonium acetate, pH 4.95, as solvent A and methanol as solvent B and a flow-rate of 0.4 mL/min (Table S2). Data was acquired at 2 Hz in negative ionization mode.

Raw metabolite data was subject to automatic intra-run calibration with sodium formate and putative metabolites identified using the Find Molecular Features peak picking software (Bruker Daltonics). Principal Component Analysis (PCA; with Pareto scaling) was performed in R, and and T-tests were performed on ProfileAnalysis software (Bruker). Only features that were detected in at least 50% of samples of both group types were used, with the exception of features that were completely undetected in one group type and detected in at least 50% of the other (which we refer to as cases of “extreme regulation”). Integrated peak signals were normalized by the sum of the feature values in the analysis.

Pathway Analysis, a combination of quantitative enrichment analysis and topology analysis, was carried out on MetaboAnalyst online software using characterized KEGG pathways for *D. melanogaster* as the back-end knowledge^95^,^119^. Enrichment analysis is based on concentration values of compounds loaded into the analysis and uses a Global Test^120^ to determine if pathways are significantly altered by an experimental condition. Topology analysis gauges the potential impact metabolite concentrations have on a particular pathway based on where the metabolites fall in its structure. Topology in our analysis was determined with a relative-betweeness centrality and normalized to the sum of the calculated importance of the pathway^95^,^119^.

## Additional Information

**How to cite this article**: MacMillan, H. A. *et al.* Cold acclimation wholly reorganizes the *Drosophila melanogaster* transcriptome and metabolome. *Sci. Rep.* **6**, 28999; doi: 10.1038/srep28999 (2016).

## Supplementary Material

Supplementary Information

Supplementary Dataset 1

Supplementary Dataset 2

Supplementary Dataset 3

Supplementary Dataset 4

## References

[b1] Overgaard J., Kearney M. R. & Hoffmann A. A. Sensitivity to thermal extremes in Australian *Drosophila* implies similar impacts of climate change on the distribution of widespread and tropical species. Glob. Chang. Biol. 20, 1738–1750 (2014). 24549716 10.1111/gcb.12521

[b2] Andersen J. L. *et al.* How to assess *Drosophila* cold tolerance: chill coma temperature and lower lethal temperature are the best predictors of cold distribution limits. Funct. Ecol. 29, 55–65 (2015).

[b3] Warren R. J. & Chick L. Upward ant distribution shift corresponds with minimum, not maximum, temperature tolerance. Glob. Chang. Biol. 2082–2088, doi: 10.1111/gcb.12169 (2013). 23504958

[b4] Parmesan C. & Yohe G. A globally coherent fingerprint of climate change impacts across natural systems. Nature 421, 37–42 (2003). 12511946 10.1038/nature01286

[b5] MacMillan H. A. *et al.* Parallel ionoregulatory adjustments underlie phenotypic plasticity and evolution of *Drosophila* cold tolerance. J. Exp. Biol. 218, 423–432 (2015). 25524989 10.1242/jeb.115790

[b6] Overgaard J., Sørensen J. G., Com E. & Colinet H. The rapid cold hardening response of *Drosophila melanogaster*: Complex regulation across different levels of biological organization. J. Insect Physiol. 62, 46–53 (2014). 24508557 10.1016/j.jinsphys.2014.01.009

[b7] Armstrong G. A. B., Rodríguez E. C. & Robertson R. M. Cold hardening modulates K^+^ homeostasis in the brain of *Drosophila melanogaster* during chill coma. J. Insect Physiol. 58, 1511–1516 (2012). 23017334 10.1016/j.jinsphys.2012.09.006

[b8] Gerken A. R., Eller O. C., Hahn D. a. & Morgan T. J. Constraints, independence, and evolution of thermal plasticity: Probing genetic architecture of long- and short-term thermal acclimation. Proc. Natl. Acad. Sci. 112, 4399–4404 (2015). 25805817 10.1073/pnas.1503456112 PMC4394312

[b9] Ransberry V. E., MacMillan H. A. & Sinclair B. J. The relationship between chill-coma onset and recovery at the extremes of the thermal window of *Drosophila melanogaster*. Physiol. Biochem. Zool. 84, 553–559 (2011). 22030848 10.1086/662642

[b10] Kelty J. Rapid cold-hardening of *Drosophila melanogaster* in a field setting. Physiol. Entomol. 32, 343–350 (2007).

[b11] Mackay T. F. C. *et al.* The *Drosophila melanogaster* genetic reference panel. Nature 482, 173–178 (2012). 22318601 10.1038/nature10811 PMC3683990

[b12] Morgan T. J. & Mackay T. F. C. Quantitative trait loci for thermotolerance phenotypes in *Drosophila melanogaster*. Heredity (Edinb). 96, 232–42 (2006). 16404413 10.1038/sj.hdy.6800786

[b13] Markow T. A. & O’Grady P. M. Drosophila biology in the genomic age. Genetics 177, 1269–76 (2007). 18039866 10.1534/genetics.107.074112 PMC2147954

[b14] Mellanby K. Low temperature and insect activity. Proc. R. Soc. B 127, 473–487 (1939).

[b15] Baust J. G. & Rojas R. R. Insect cold hardiness: Facts and fancy. J. Insect Physiol. 31, 755–759 (1985).

[b16] Hazell S. P. & Bale J. S. Low temperature thresholds: are chill coma and CTmin synonymous? J. Insect Physiol. 57, 1085–1089 (2011). 21510951 10.1016/j.jinsphys.2011.04.004

[b17] MacMillan H. A. & Sinclair B. J. Mechanisms underlying insect chill-coma. J. Insect Physiol. 57, 12–20 (2011). 20969872 10.1016/j.jinsphys.2010.10.004

[b18] MacMillan H. A., Andersen J. L., Loeschcke V. & Overgaard J. Sodium distribution predicts the chill tolerance of *Drosophila melanogaster* raised in different thermal conditions. Am. J. Physiol. Regul. Integr. Comp. Physiol. 308, 823–831 (2015). 10.1152/ajpregu.00465.2014 25761700

[b19] Yi S.-X., Moore C. W. & Lee R. E. J. Rapid cold-hardening protects *Drosophila melanogaster* from cold-induced apoptosis. Apoptosis 12, 1183–1193 (2007). 17245639 10.1007/s10495-006-0048-2

[b20] David R. J. *et al.* Cold stress tolerance in *Drosophila*: analysis of chill coma recovery in D. melanogaster. J. Therm. Biol. 23, 291–299 (1998).

[b21] Gilchrist G. W., Huey R. B. & Partridge L. Thermal sensitivity of *Drosophila melanogaster*: evolutionary responses of adults and eggs to laboratory natural selection at different temperatures. Physiol. Zool. 70, 403–14 (1997). 9237300 10.1086/515853

[b22] Anderson A., Hoffmann A. A. & McKechnie S. W. Response to selection for rapid chill-coma recovery in *Drosophila melanogaster*: physiology and life-history traits. Genet. Res. 85, 15–22 (2005). 16089033 10.1017/s0016672304007281

[b23] Hoffmann A. A. Physiological climatic limits in *Drosophila*: patterns and implications. J. Exp. Biol. 213, 870–880 (2010). 20190112 10.1242/jeb.037630

[b24] Goto S. G. & Kimura M. T. Heat- and cold-shock responses and temperature adaptations in subtropical and temperate species of *Drosophila*. J. Insect Physiol. 44, 1233–1239 (1998). 12770323 10.1016/s0022-1910(98)00101-2

[b25] Kellermann V. *et al.* Phylogenetic constraints in key functional traits behind species’ climate niches: patterns of desiccation and cold resistance across 95 *Drosophila* species. Evolution 66, 3377–3389 (2012). 23106704 10.1111/j.1558-5646.2012.01685.x

[b26] Gibert P. & Huey R. B. Chill-coma temperature in *Drosophila*: Effects of developmental temperature, latitude, and phylogeny. Physiol. Biochem. Zool. 74, 429–434 (2001). 11331516 10.1086/320429

[b27] Overgaard J., Kristensen T. N., Mitchell K. A. & Hoffmann A. A. Thermal tolerance in widespread and tropical *Drosophila* species: does phenotypic plasticity increase with latitude? Am. Nat. 178, S80–S96 (2011). 21956094 10.1086/661780

[b28] MacMillan H. A., Andersen J. L., Davies S. A. & Overgaard J. The capacity to maintain ion and water homeostasis underlies interspecific variation in *Drosophila* cold tolerance. Sci. Rep. 5, 18607 (2015). 26678786 10.1038/srep18607 PMC4683515

[b29] Cooper B. S., Hammad L. a. & Montooth K. L. Thermal adaptation of cellular membranes in natural populations of *Drosophila melanogaster*. Funct. Ecol. 28, 886–894 (2014). 25382893 10.1111/1365-2435.12264 PMC4219941

[b30] Slotsbo S. *et al.* Tropical to sub-polar gradient in phospholipid composition suggests adaptive tuning of biological membrane function in drosophilids. Funct. Ecol., doi: 10.1111/1365-2435.12568 (2015).

[b31] Williams C. M. *et al.* Cold adaptation shapes the robustness of metabolic networks in *Drosophila melanogaster*. Evolution 68, 3505–23 (2014). 25308124 10.1111/evo.12541 PMC4472466

[b32] Norry F. M., Gomez F. H. & Loeschcke V. Knockdown resistance to heat stress and slow recovery from chill coma are genetically associated in a quantitative trait locus region of chromosome 2 in *Drosophila melanogaster*. Mol. Ecol. 16, 3274–84 (2007). 17651203 10.1111/j.1365-294X.2007.03335.x

[b33] Norry F. M., Scannapieco A. C., Sambucetti P., Bertoli C. I. & Loeschcke V. QTL for the thermotolerance effect of heat hardening, knockdown resistance to heat and chill-coma recovery in an intercontinental set of recombinant inbred lines of *Drosophila melanogaster*. Mol. Ecol. 17, 4570–81 (2008). 18986501 10.1111/j.1365-294X.2008.03945.x

[b34] Qin W., Neal S. J., Robertson R. M., Westwood J. T. & Walker V. K. Cold hardening and transcriptional change in *Drosophila melanogaster*. Insect Mol. Biol. 14, 607–13 (2005). 16313561 10.1111/j.1365-2583.2005.00589.x

[b35] Zhang J., Marshall K. E., Westwood J. T., Clark M. S. & Sinclair B. J. Divergent transcriptomic responses to repeated and single cold exposures in *Drosophila melanogaster*. J. Exp. Biol. 214, 4021–9 (2011). 22071194 10.1242/jeb.059535

[b36] Colinet H., Overgaard J., Com E. & Sørensen J. G. Proteomic profiling of thermal acclimation in *Drosophila melanogaster*. Insect Biochem. Mol. Biol. 43, 352–65 (2013). 23416132 10.1016/j.ibmb.2013.01.006

[b37] Bing X., Zhang J. & Sinclair B. J. A comparison of *Frost* expression among species and life stages of *Drosophila*. Insect Mol. Biol. 21, 31–9 (2012). 21955087 10.1111/j.1365-2583.2011.01108.x

[b38] Colinet H., Lee S. F. & Hoffmann A. Functional characterization of the *Frost* gene in *Drosophila melanogaster*: importance for recovery from chill coma. PLos One 5, e10925 (2010). 20532197 10.1371/journal.pone.0010925 PMC2880008

[b39] Udaka H., Ueda C. & Goto S. G. Survival rate and expression of *Heat-shock protein 70* and *Frost* genes after temperature stress in *Drosophila melanogaster* lines that are selected for recovery time from temperature coma. J. Insect Physiol. 56, 1889–1894 (2010). 20713057 10.1016/j.jinsphys.2010.08.008

[b40] Goto M., Li Y. & Honma T. Changes of diapause and cold hardiness in the Shonai ecotype larvae of the rice stem borer, *Chilo suppressalis* Walker (Lepidoptera: Pyralidae) during overwintering. Appl. Entomol. Zool. 36, 323–328 (2001).

[b41] Sinclair B. J., Gibbs A. G. & Roberts S. P. Gene transcription during exposure to, and recovery from, cold and desiccation stress in *Drosophila melanogaster*. Insect Mol. Biol. 16, 435–443 (2007). 17506850 10.1111/j.1365-2583.2007.00739.x

[b42] Rako L., Blacket M. J., McKechnie S. W. & Hoffmann A. A. Candidate genes and thermal phenotypes: identifying ecologically important genetic variation for thermotolerance in the Australian *Drosophila melanogaster* cline. Mol. Ecol. 16, 2948–2957 (2007). 17614909 10.1111/j.1365-294X.2007.03332.x

[b43] Udaka H., Percival-Smith A. & Sinclair B. J. Increased abundance of *Frost* mRNA during recovery from cold stress is not essential for cold tolerance in adult *Drosophila melanogaster*. Insect Mol. Biol. 22, 541–50 (2013). 23901849 10.1111/imb.12044

[b44] Teets N. M., Yi S.-X., Lee R. E. & Denlinger D. L. Calcium signaling mediates cold sensing in insect tissues. Proc. Natl. Acad. Sci. USA 110, 9154–9159 (2013). 23671084 10.1073/pnas.1306705110 PMC3670363

[b45] Colinet H. & Hoffmann A. A. Comparing phenotypic effects and molecular correlates of developmental, gradual and rapid cold acclimation responses in *Drosophila melanogaster*. Funct. Ecol. 26, 84–93 (2012).

[b46] Dennis A. B., Dunning L. T., Sinclair B. J. & Buckley T. R. Parallel molecular routes to cold adaptation in eight genera of New Zealand stick insects. Sci. Rep. 5, 13965 (2015). 26355841 10.1038/srep13965 PMC4564816

[b47] Dunning L. T. *et al.* Identification of cold-responsive genes in a New Zealand alpine stick insect using RNA-Seq. Comp. Biochem. Physiol. D 8, 24–31 (2013). 10.1016/j.cbd.2012.10.005 23220720

[b48] Parker D. J. *et al.* How consistent are the transcriptome changes associated with cold acclimation in two species of the *Drosophila virilis* group? Heredity (Edinb). 115, 13–21 (2015). 25669607 10.1038/hdy.2015.6 PMC4815502

[b49] Sulmon C. *et al.* Abiotic stressors and stress responses: What commonalities appear between species across biological organization levels? Environ. Pollut. 202, 66–77 (2015). 25813422 10.1016/j.envpol.2015.03.013

[b50] Findsen A., Pedersen T. H., Petersen A. G., Nielsen O. B. & Overgaard J. Why do insects enter and recover from chill coma? Low temperature and high extracellular potassium compromise muscle function in *Locusta migratoria*. J. Exp. Biol. 217, 1297–1306 (2014). 24744424 10.1242/jeb.098442

[b51] Langfeld K. S., Crockford T. & Johnston I. A. Temperature acclimation in the common carp: Force-velocity characteristics and myosin subunit composition of slow muscle fibres. J. Exp. Biol. 155, 291–304 (1991).

[b52] Scott G. R. & Johnston I. A. Temperature during embryonic development has persistent effects on thermal acclimation capacity in zebrafish. Proc. Natl. Acad. Sci. USA 109, 14247–52 (2012). 22891320 10.1073/pnas.1205012109 PMC3435178

[b53] Gracey A. Y. *et al.* Coping with cold: An integrative, multitissue analysis of the transcriptome of a poikilothermic vertebrate. Proc. Natl. Acad. Sci. USA 101, 16970–5 (2004). 15550548 10.1073/pnas.0403627101 PMC534716

[b54] Andersen J. L., MacMillan H. A. & Overgaard J. Muscle membrane potential and insect chill coma. J. Exp. Biol. 218, 2492–2495 (2015). 26089529 10.1242/jeb.123760

[b55] Hosler J. S., Burns J. E. & Esch H. E. Flight muscle resting potential and species-specific differences in chill-coma. J. Insect Physiol. 46, 621–627 (2000). 10742510 10.1016/s0022-1910(99)00148-1

[b56] Frazier M. R., Harrison J. F., Kirkton S. D. & Roberts S. P. Cold rearing improves cold-flight performance in *Drosophila* via changes in wing morphology. J. Exp. Biol. 211, 2116–2122 (2008). 18552301 10.1242/jeb.019422

[b57] Johnston I. A. Calcium regulatory proteins and temperature acclimation of actomyosin ATPase from a eurythermal teleost (*Carassius auratus* L.). J. Comp. Physiol. 129, 163–167 (1979).

[b58] Kim M., Robich R. M., Rinehart J. P. & Denlinger D. L. Upregulation of two actin genes and redistribution of actin during diapause and cold stress in the northern house mosquito, Culex pipiens. J. Insect Physiol. 52, 1226–1233 (2006). 17078965 10.1016/j.jinsphys.2006.09.007 PMC1839883

[b59] Cottam D. M. *et al.* Non-centrosomal microtubule-organising centres in cold-treated cultured *Drosophila* cells. Cell Motil. Cytoskeleton 63, 88–100 (2006). 16385467 10.1002/cm.20103

[b60] Goto S. Expression of *Drosophila* homologue of senescence marker protein-30 during cold acclimation. J. Insect Physiol. 46, 1111–1120 (2000). 10817837 10.1016/s0022-1910(99)00221-8

[b61] Clowers K. J., Lyman R. F., Mackay T. F. C. & Morgan T. J. Genetic variation in senescence marker protein-30 is associated with natural variation in cold tolerance in *Drosophila*. Genet. Res. (Camb). 92, 103–113 (2010). 20515514 10.1017/S0016672310000108

[b62] Collinge J. E., Anderson A. R., Weeks A. R., Johnson T. K. & McKechnie S. W. Latitudinal and cold-tolerance variation associate with DNA repeat-number variation in the hsr-omega RNA gene of *Drosophila melanogaster*. Heredity (Edinb). 101, 260–70 (2008). 18560441 10.1038/hdy.2008.57

[b63] Colinet H., Siaussat D., Bozzolan F. & Bowler K. Rapid decline of cold tolerance at young age is associated with expression of stress genes in *Drosophila melanogaster*. J. Exp. Biol. 216, 253–9 (2013). 22996448 10.1242/jeb.076216

[b64] Colinet H. & Hoffmann A. Gene and protein expression of *Drosophila* Starvin during cold stress and recovery from chill coma. Insect Biochem. Mol. Biol. 40, 425–428 (2010). 20303406 10.1016/j.ibmb.2010.03.002

[b65] Štětina T., Koštál V. & Korbelová J. The role of inducible Hsp70, and other heat shock proteins, in adaptive complex of cold tolerance of the fruit fly (*Drosophila melanogaster*). PLos One 10, e0128976 (2015). 26034990 10.1371/journal.pone.0128976 PMC4452724

[b66] Chintapalli V. R., Wang J. & Dow J. A. T. Using FlyAtlas to identify better *Drosophila melanogaster* models of human disease. Nat. Genet. 39, 715–720 (2007). 17534367 10.1038/ng2049

[b67] Terhzaz S. *et al.* Insect capa neuropeptides impact desiccation and cold tolerance. Proc. Natl. Acad. Sci. 112, 2882–2887 (2015). 25730885 10.1073/pnas.1501518112 PMC4352776

[b68] MacMillan H. A., Williams C. M., Staples J. F. & Sinclair B. J. Reestablishment of ion homeostasis during chill-coma recovery in the cricket *Gryllus pennsylvanicus*. Proc. Natl. Acad. Sci. USA 109, 20750–20755 (2012). 23184963 10.1073/pnas.1212788109 PMC3528563

[b69] Kelkenberg M., Odman-Naresh J., Muthukrishnan S. & Merzendorfer H. Chitin is a necessary component to maintain the barrier function of the peritrophic matrix in the insect midgut. Insect Biochem. Mol. Biol. 56, 21–28 (2015). 25449129 10.1016/j.ibmb.2014.11.005

[b70] MacMillan H. A. & Sinclair B. J. The role of the gut in insect chilling injury: cold-induced disruption of osmoregulation in the fall field cricket, Gryllus pennsylvanicus. J. Exp. Biol. 214, 726–734 (2011). 21307058 10.1242/jeb.051540

[b71] Vermeulen C. J., Sørensen P., Kirilova Gagalova K. & Loeschcke V. Transcriptomic analysis of inbreeding depression in cold-sensitive *Drosophila melanogaster* shows upregulation of the immune response. J. Evol. Biol. 26, 1890–902 (2013). 23944235 10.1111/jeb.12183

[b72] Xu J. & James R. R. Temperature stress affects the expression of immune response genes in the alfalfa leafcutting bee, Megachile rotundata. Insect Mol. Biol. 21, 269–280 (2012). 22356318 10.1111/j.1365-2583.2012.01133.x

[b73] Sinclair B. J., Ferguson L. V., Salehipour-shirazi G. & MacMillan H. A. Cross-tolerance and cross-talk in the cold: relating low temperatures to desiccation and immune stress in insects. Integr. Comp. Biol. 53, 545–556 (2013). 23520401 10.1093/icb/ict004

[b74] Ferguson L. V., Heinrichs D. E. & Sinclair B. J. Paradoxical acclimation responses in the thermal performance of insect immunity. Oecologia 181, 77–85 (2016). 26846428 10.1007/s00442-015-3529-6

[b75] Roberts D. B. & Brock H. W. The major serum proteins of Dipteran larvae. Experientia 37, 103–110 (1981).

[b76] Poupardin R. *et al.* Early transcriptional events linked to induction of diapause revealed by RNAseq in larvae of drosophilid fly, Chymomyza costata. BMC Genomics 16, 720 (2015). 26391666 10.1186/s12864-015-1907-4 PMC4578651

[b77] Overgaard J. *et al.* Metabolomic profiling of rapid cold hardening and cold shock in *Drosophila melanogaster*. J. Insect Physiol. 53, 1218–1232 (2007). 17662301 10.1016/j.jinsphys.2007.06.012

[b78] Koštál V. *et al.* Long-term cold acclimation extends survival time at 0 °C and modifies the metabolomic profiles of the larvae of the fruit fly *Drosophila melanogaster*. PLos One 6, e25025 (2011). 21957472 10.1371/journal.pone.0025025 PMC3177886

[b79] Colinet H., Larvor V., Laparie M. & Renault D. Exploring the plastic response to cold acclimation through metabolomics. Funct. Ecol. 26, 711–722 (2012).

[b80] Teets N. M. & Denlinger D. L. Physiological mechanisms of seasonal and rapid cold-hardening in insects. Physiol. Entomol. 38, 105–116 (2013).

[b81] Koštál V., Vambera J. & Bastl J. On the nature of pre-freeze mortality in insects: water balance, ion homeostasis and energy charge in the adults of *Pyrrhocoris apterus*. J. Exp. Biol. 207, 1509–1521 (2004). 15037645 10.1242/jeb.00923

[b82] MacMillan H. A., Williams C. M., Staples J. F. & Sinclair B. J. Metabolism and energy supply below the critical thermal minimum of a chill-susceptible insect. J. Exp. Biol. 215, 1366–1372 (2012). 22442375 10.1242/jeb.066381

[b83] Colinet H. Disruption of ATP homeostasis during chronic cold stress and recovery in the chill susceptible beetle (*Alphitobius diaperinus*). Comp. Biochem. Physiol. A 160, 63–7 (2011). 10.1016/j.cbpa.2011.05.003 21596153

[b84] Dollo V. H., Yi S.-X. & Lee R. E. High temperature pulses decrease indirect chilling injury and elevate ATP levels in the flesh fly, Sarcophaga crassipalpis. Cryobiology 60, 351–353 (2010). 20233586 10.1016/j.cryobiol.2010.03.002

[b85] Saunders D. S., Richard D. S., Applebaum S. W., Ma M. & Gilbert L. I. Photoperiodic diapause in *Drosophila melanogaster* involves a block to the juvenile hormone regulation of ovarian maturation. Gen. Comp. Endocrinol. 79, 174–184 (1990). 2118114 10.1016/0016-6480(90)90102-r

[b86] Wijesekera T. P., Saurabh S. & Dauwalder B. Juvenile Hormone Is Required in Adult Males for Drosophila Courtship. PLos One 11, e0151912 (2016). 27003411 10.1371/journal.pone.0151912 PMC4803231

[b87] Horwath K. L. & Duman J. G. Induction of antifreeze protein production by juvenile hormone in larvae of the beetle, Dendroides canadensis. J. Comp. Physiol. B 151, 233–240 (1983).

[b88] Toxopeus J., Jakobs R., Ferguson L. V., Gariepy T. D. & Sinclair B. J. Reproductive arrest and stress resistance in winter-acclimated Drosophila suzukii. J. Insect Physiol. 89, 37–51 (2016). 27039032 10.1016/j.jinsphys.2016.03.006

[b89] Chintapalli V. R. *et al.* Transport proteins NHA1 and NHA2 are essential for survival, but have distinct transport modalities. Proc. Natl. Acad. Sci. 112, 11720–11725 (2015). 26324901 10.1073/pnas.1508031112 PMC4577160

[b90] Bang S. *et al.* Dopamine signalling in mushroom bodies regulates temperature-preference behaviour in *Drosophila*. PLos Genet. 7, (2011). 10.1371/journal.pgen.1001346 PMC3063753 21455291

[b91] Ueno T., Tomita J., Kume S. & Kume K. Dopamine modulates metabolic rate and temperature sensitivity in *Drosophila melanogaster*. PLos One 7, e31513 (2012). 22347491 10.1371/journal.pone.0031513 PMC3274542

[b92] Yamamoto S. & Seto E. S. Dopamine dynamics and signaling in *Drosophila*: An overview of genes, drugs and behavioral paradigms. Exp. Anim. 63, 107–119 (2014). 24770636 10.1538/expanim.63.107 PMC4160991

[b93] Crill W. D., Huey R. B. & Gilchrist G. W. Within- and between-generation effects of temperature on the morphology and physiology of *Drosophila melanogaster*. Evolution 50, 1205–1218 (1996). 10.1111/j.1558-5646.1996.tb02361.x 28565273

[b94] Kutch I. C., Sevgili H., Wittman T. & Fedorka K. M. Thermoregulatory strategy may shape immune investment in *Drosophila melanogaster*. J. Exp. Biol. 217, 3664–9 (2014). 25147243 10.1242/jeb.106294

[b95] Xia J., Mandal R., Sinelnikov I. V., Broadhurst D. & Wishart D. S. MetaboAnalyst 2.0-a comprehensive server for metabolomic data analysis. Nucleic Acids Res. 40, W127–W133 (2012). 22553367 10.1093/nar/gks374 PMC3394314

[b96] Young M. D., Wakefield M. J., Smyth G. K. & Oshlack A. Gene ontology analysis for RNA-seq: accounting for selection bias. Genome Biol. 11, R14 (2010). 20132535 10.1186/gb-2010-11-2-r14 PMC2872874

[b97] Misener S. R., Chen C.-P. & Walker V. K. Cold tolerance and proline metabolic gene expression in *Drosophila melanogaster*. J. Insect Physiol. 47, 393–400 (2001). 11166304 10.1016/s0022-1910(00)00141-4

[b98] Koštál V., Šimek P., Zahradníčková H., Cimlová J. & Štětina T. Conversion of the chill susceptible fruit fly larva (*Drosophila melanogaster*) to a freeze tolerant organism. Proc. Natl. Acad. Sci. USA 109, 3270–3274 (2012). 22331891 10.1073/pnas.1119986109 PMC3295325

[b99] Yancey P. H. Organic osmolytes as compatible, metabolic and counteracting cytoprotectants in high osmolarity and other stresses. J. Exp. Biol. 208, 2819–30 (2005). 16043587 10.1242/jeb.01730

[b100] Krishnan N., Dickman M. B. & Becker D. F. Proline modulates the intracellular redox environment and protects mammalian cells against oxidative stress. Free Radic. Biol. Med. 44, 671–81 (2008). 18036351 10.1016/j.freeradbiomed.2007.10.054 PMC2268104

[b101] Lalouette L., Williams C. M., Hervant F., Sinclair B. J. & Renault D. Metabolic rate and oxidative stress in insects exposed to low temperature thermal fluctuations. Comp. Biochem. Physiol. A 158, 229–34 (2011). 10.1016/j.cbpa.2010.11.007 21074633

[b102] Rojas R. R. & Leopold R. A. Chilling injury in the housefly: evidence for the role of oxidative stress between pupariation and emergence. Cryobiology 33, 447–458 (1996).

[b103] Joanisse D. & Storey K. Oxidative stress and antioxidants in overwintering larvae of cold-hardy goldenrod gall insects. J. Exp. Biol. 199, 1483–91 (1996). 9319381 10.1242/jeb.199.7.1483

[b104] Grubor-Lajsic G. *et al.* Effect of cold acclimation on the antioxidant defense system of two larval Lepidoptera (Noctuidae). Arch. Insect Biochem. Physiol. 36, 1–10 (1997).

[b105] Shang Q. *et al.* Proteomics analysis of overexpressed plasma proteins in response to cold acclimation in *Ostrinia furnacalis*. Arch. Insect Biochem. Physiol. 90, 195–208 (2015). 26440752 10.1002/arch.21302

[b106] Kocsy G., Galiba G. & Brunold C. Role of glutathione in adaptation and signalling during chilling and cold acclimation in plants. Physiol. Plant. 113, 158–164 (2001). 12060292 10.1034/j.1399-3054.2001.1130202.x

[b107] Hayward S. A. L., Manso B. & Cossins A. R. Molecular basis of chill resistance adaptations in poikilothermic animals. J. Exp. Biol. 217, 6–15 (2014). 24353199 10.1242/jeb.096537

[b108] Overgaard J. *et al.* Effects of acclimation temperature on thermal tolerance and membrane phospholipid composition in the fruit fly *Drosophila melanogaster*. J. Insect Physiol. 54, 619–629 (2008). 18280492 10.1016/j.jinsphys.2007.12.011

[b109] Marshall K. E. & Sinclair B. J. Repeated stress exposure results in a survival-reproduction trade-off in *Drosophila melanogaster*. Proc. R. Soc. B 277, 963–969 (2010). 10.1098/rspb.2009.1807 PMC2842730 19939842

[b110] Nilson T. L., Sinclair B. J. & Roberts S. P. The effects of carbon dioxide anesthesia and anoxia on rapid cold-hardening and chill coma recovery in *Drosophila melanogaster*. J. Insect Physiol. 52, 1027–1033 (2006). 16996534 10.1016/j.jinsphys.2006.07.001 PMC2048540

[b111] Goecks J., Nekrutenko A., Taylor J. & Team T. G. Galaxy: a comprehensive approach for supporting accessible, reproducible, and transparent computational research in the life sciences. Genome Biol. 11, R86 (2010). 20738864 10.1186/gb-2010-11-8-r86 PMC2945788

[b112] Trapnell C. *et al.* Differential gene and transcript expression analysis of RNA-seq experiments with TopHat and Cufflinks. Nat. Protoc. 7, 562–578 (2012). 22383036 10.1038/nprot.2012.016 PMC3334321

[b113] Trapnell C., Pachter L. & Salzberg S. L. TopHat: discovering splice junctions with RNA-Seq. Bioinformatics 25, 1105–1111 (2009). 19289445 10.1093/bioinformatics/btp120 PMC2672628

[b114] Trapnell C. *et al.* Transcript assembly and quantification by RNA-Seq reveals unannotated transcripts and isoform switching during cell differentiation. Nat. Biotechnol. 28, 511–515 (2010). 20436464 10.1038/nbt.1621 PMC3146043

[b115] Benjamini Y. & Hochberg Y. Controlling the false discuvery rate: a practical and powerful approach to multiple testing. J. R. Stat. Soc. Ser. B 57, 289–300 (1995).

[b116] Trapnell C. *et al.* Differential analysis of gene regulation at transcript resolution with RNA-seq. Nat. Biotechnol. 31, 46–53 (2013). 23222703 10.1038/nbt.2450 PMC3869392

[b117] Supek F., Bošnjak M., Škunca N. & Šmuc T. REVIGO summarizes and visualizes long lists of gene ontology terms. PLos One 6, e21800 (2011). 21789182 10.1371/journal.pone.0021800 PMC3138752

[b118] Knee J. M., Rzezniczak T. Z., Barsch A., Guo K. Z. & Merritt T. J. S. A novel ion pairing LC/MS metabolomics protocol for study of a variety of biologically relevant polar metabolites. J. Chromatogr. B. Analyt. Technol. Biomed. Life Sci. 936, 63–73 (2013). 10.1016/j.jchromb.2013.07.027 24004912

[b119] Xia J., Psychogios N., Young N. & Wishart D. S. MetaboAnalyst: a web server for metabolomic data analysis and interpretation. Nucleic Acids Res. 37, W652–W660 (2009). 19429898 10.1093/nar/gkp356 PMC2703878

[b120] Goeman J. J. & Bühlmann P. Analyzing gene expression data in terms of gene sets: methodological issues. Bioinformatics 23, 980–987 (2007). 17303618 10.1093/bioinformatics/btm051

