# Supplementary material for: Cold acclimation wholly reorganizes the Drosophila melanogaster transcriptome and metabolome
Source: Sci Rep. 2016 Jun 30;6:28999. doi: 10.1038/srep28999 (PMC4928047; doi:10.1038/srep28999)
Supplement: Supplementary Information [file srep28999-s1.pdf]

# Supplementary Material

## Cold acclimation wholly reorganizes the *Drosophila melanogaster* transcriptome and metabolome

Heath A. MacMillan[1*†], Jose M. Knee[2†], Alice B. Dennis[3,4†], Hiroko Udaka[1†], Katie E. Marshall[1†], Thomas J. S. Merritt[2], and Brent J. Sinclair[1]

1. Department of Biology, University of Western Ontario, London, ON, Canada
2. Department of Chemistry and Biochemistry, Laurentian University, Sudbury, ON, Canada
3. Landcare Research, Auckland, New Zealand.
4. Allan Wilson Centre for Molecular Ecology and Evolution, Auckland, New Zealand

* - Corresponding author
† - Present addresses: **HAM**: Department of Biology, York University, Toronto, Canada
**JMK**: Advanced Medical Research Institute of Canada, Sudbury, Canada
**ABD**: Institute of Integrative Biology, ETH Zürich, Zürich, Switzerland; EAWAG, Swiss Federal Institute of Aquatic Science and Technology, Dübendorf, Switzerland
**HU**: Department of Zoology, Kyoto University, Kyoto, Japan.
**KEM**: Department of Zoology, University of British Colombia, Vancouver, Canada

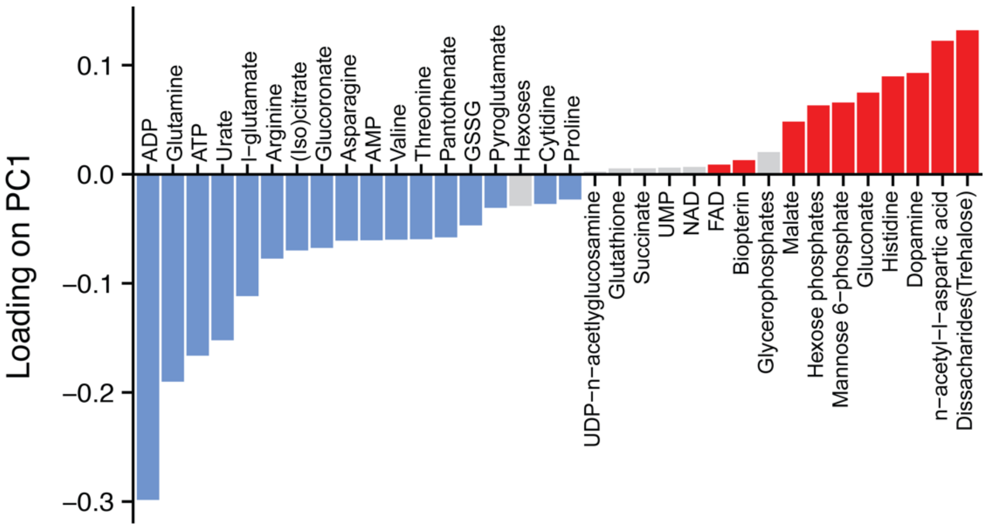


**Figure S1. Extended results of principal components analysis of metabolite abundance in whole-body homogenates of *D. melanogaster* males, acclimated as adults to 21.5°C or 6°C.** Loadings of identified metabolites along principal component 1 (PC1). Metabolites that were significantly more abundant in cold-acclimated flies or warm acclimated flies are shown as blue or red bars, respectively. Metabolites that did not differ in abundance between the acclimation groups are shown as grey bars.

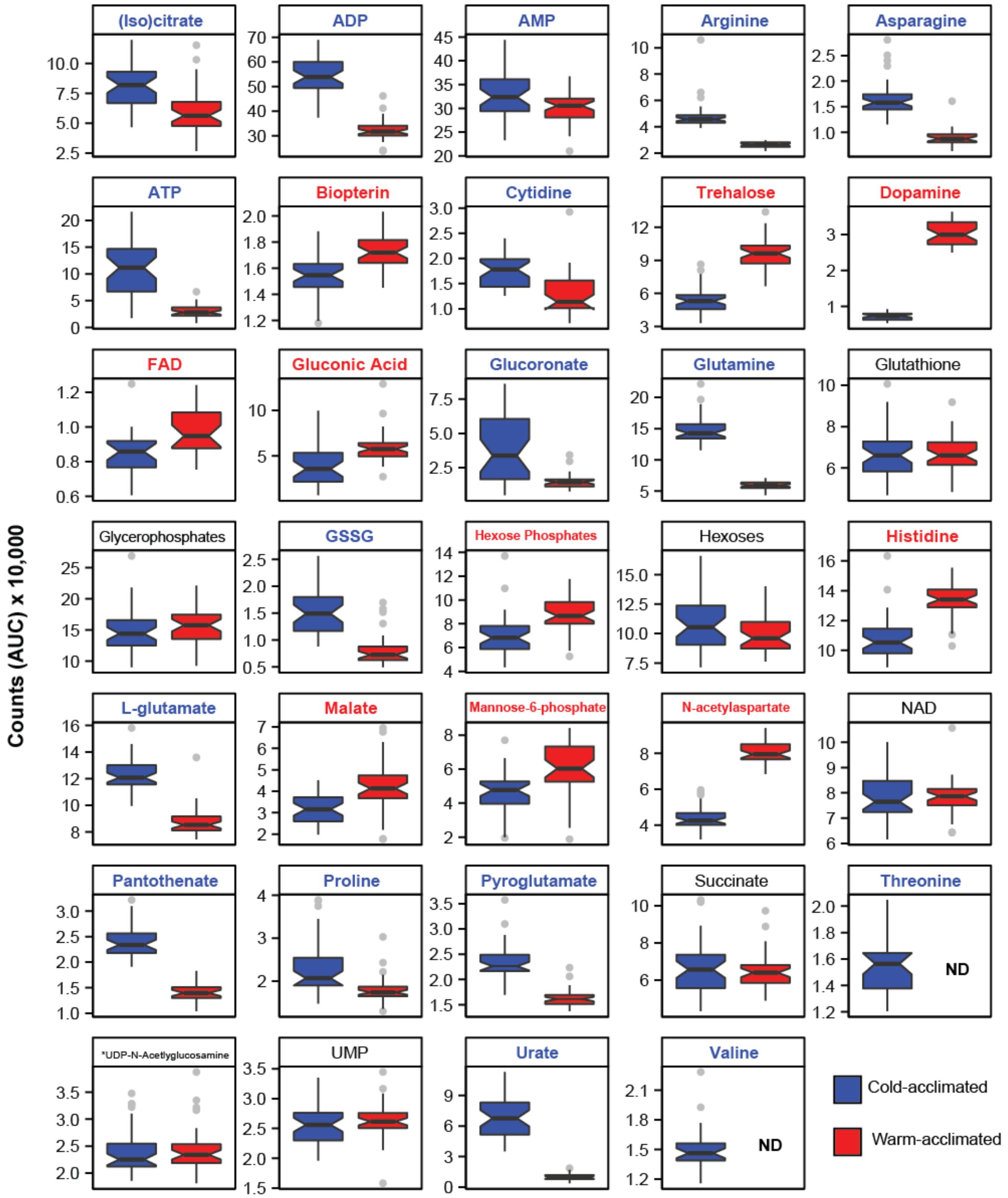


**Figure S2: Boxplots of metabolite peak areas from whole-body homogenates of male *D. melanogaster* acclimated to 21.5°C (red) and 6°C (blue) as adults.** Names of metabolites that were significantly more abundant in warm- or cold-acclimated flies are shown in red or blue, respectively. ND: not detected. Metabolite abundance is expressed as area under the curve (AUC). Notches in boxplots represent 95% confidence intervals.

**Table S1.** Metabolites detected in LC-MS analysis. Metabolites in bold typeface were significantly higher in warm- (red) or cold-acclimated (blue) flies following false discovery rate (FDR) correction.

| Metabolite | *P*-value | Fold Change (Cold/Warm) | *Q*-value (FDR) |
|---|---|---|---|
| **Threonine** | **Undetected in warm acclimated flies** | | |
| **Valine** | **Undetected in warm acclimated flies** | | |
| **Urate** | **<0.0001** | **6.67** | **<0.00001** |
| **Adenosine 5'-triphosphate (ATP)** | **<0.0001** | **3.57** | **<0.00001** |
| **Glucoronate** | **0.00001** | **2.56** | **0.00002** |
| **Glutamine** | **<0.0001** | **2.50** | **<0.00001** |
| **Oxidized Glutathione (GSSG)** | **<0.0001** | **1.92** | **<0.00001** |
| **Asparagine** | **<0.0001** | **1.82** | **<0.00001** |
| **Arginine** | **<0.0001** | **1.82** | **<0.00001** |
| **Pantothenate** | **<0.0001** | **1.69** | **<0.00001** |
| **Adenosine 5'-diphosphate (ADP)** | **<0.0001** | **1.67** | **<0.00001** |
| **Pyroglutamate** | **<0.0001** | **1.45** | **<0.00001** |
| **Glutamate** | **<0.0001** | **1.39** | **<0.00001** |
| **Cytidine** | **0.00001** | **1.37** | **0.00001** |
| **(Iso)citrate** | **0.00001** | **1.35** | **0.00002** |
| **Proline** | **0.00015** | **1.27** | **0.00032** |
| Hexoses | 0.02942 | 1.10 | 0.0502 |
| **Adinosine 5'-monophosphate (AMP)** | **0.00469** | **1.09** | **0.00872** |
| Nicotinamide adenine dinucleotide (NAD) | 0.50884 | 1.02 | 0.59304 |
| Succinate | 0.55667 | 1.02 | 0.62749 |
| UDP-n-acetylglucosamine (UDP-glcNac) | 0.81817 | -1.01 | 0.83546 |
| Glutathione (GSH) | 0.87505 | -1.01 | 0.88113 |
| Uridine 5'monophosphate (UMP) | 0.29389 | -1.03 | 0.37707 |
| Glycerophosphates | 0.3377 | -1.05 | 0.41852 |
| **Biopterin** | **<0.0001** | **-1.11** | **0.00001** |
| **Flavin adenine dinucleotide (FAD)** | **0.0009** | **-1.16** | **0.00183** |
| **Histidine** | **<0.0001** | **-1.24** | **<0.00001** |
| **Hexose phosphates** | **0.00001** | **-1.25** | **0.00003** |
| **Mannose 6-phosphate** | **0.00002** | **-1.3** | **0.00006** |
| **Malate** | **0.00003** | **-1.32** | **0.00006** |
| **Gluconate** | **0.00005** | **-1.52** | **0.00011** |
| **Trehalose** | **<0.0001** | **-1.78** | **<0.00001** |
| **N-acetyl-l-aspartate (NAA)** | **<0.0001** | **-1.84** | **<0.00001** |
| **Dopamine** | **<0.0001** | **-4.25** | **<0.00001** |

**Table S2. Metabolite separation solvent table**. Metabolites were separated using a multi-step, two-solvent gradient with diamylammonium acetate, pH 4.95, as solvent A and methanol as solvent B and a flow-rate of 0.4 mL/min.

| Step | Total Time (min) | Solvent A (%) | Solvent B (%) |
|---|---|---|---|
| 1 | 1.25 | 95 | 5 |
| 2 | 5.25 | 80 | 20 |
| 3 | 15.25 | 80 | 20 |
| 4 | 17.50 | 65 | 35 |
| 5 | 19.25 | 65 | 35 |
| 6 | 21.00 | 40 | 60 |
| 7 | 22.75 | 40 | 60 |
| 8 | 22.85 | 10 | 90 |
| 9 | 24.50 | 10 | 90 |
| 10 | 24.60 | 95 | 5 |
| 11 | 31.50 | 95 | 5 |
